# Airborne infections related to virus aerosol contamination at indoor cultural venues: Recommendations on how to minimize

**DOI:** 10.1002/puh2.59

**Published:** 2023-01-20

**Authors:** Tunga Salthammer, Heinz‐Jörn Moriske

**Affiliations:** ^1^ Fraunhofer WKI Department of Material Analysis and Indoor Chemistry Braunschweig Germany; ^2^ Formerly German Federal Environment Agency Dessau‐Roßlau Germany

**Keywords:** public events, safety measures, ventilation

## Abstract

**Background:**

The COVID‐19 pandemic has resulted in many live events being canceled or held without spectator participation. As a result, a series of investigations were carried out and strategies developed to determine the requirements under which cultural activities can be maintained. This work summarizes published studies and provides recommendations for performing cultural events under pandemic conditions.

**Methods:**

The available literature search was evaluated in accordance with Preferred Reporting Items for Systematic Reviews and Meta‐Analyses. The results were combined with findings, guidelines, and regulations for conducting courses in other indoor environments under pandemic conditions, for example, school classrooms. Recommendations were derived, the consideration of which can enable the continuation of cultural events.

**Results:**

The published studies can only take into account the previous conditions of the pandemic situation with the known virus mutations. However, the number of experimental investigations including analytical and medical proof of infections, surveys, and simulations is comparatively small. This is due to the complexity of the events as well as the priority and urgency of the school issue. Cultural events take place under very different conditions. It is therefore practically impossible to predict the risk of infection for a specific situation with many potential virus spreaders attending or to derive general rules that go beyond the known measures of vaccination, testing, masks, and distance.

**Conclusion:**

Cultural events can be held under pandemic conditions provided certain requirements are met. Most study results agree on this. Any recommendations on hygiene, safety, and ventilation measures in cultural facilities under pandemic conditions can reduce the risk of infection but cannot completely eliminate it. It is also of considerable importance that visitors protect themselves individually and act responsibly.

## Graphical abstract

In this work, the risk of infection at indoor cultural events with a focus on SARS‐CoV‐2 is examined. Under certain circumstances, performances may also be held during pandemics provided that the appropriate framework conditions and recommended measures are observed.

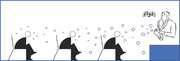

## INTRODUCTION

The cultural sectors in Europe are among the most diverse in the world. Art and culture are an integral part of society [1]. Above all, cultural events contribute to social cohesion in their function as places of encounter and participation. The COVID‐19 pandemic with the associated standstill in large parts of public life has led to major individual and societal problems [2] and made it particularly clear how much poorer our society is without the direct experience of art and culture. In view of the current situation and with a view to possible future waves of infection and pandemics, efforts must be made to make cultural events as pandemic‐proof as possible [3]. The operation of opera houses, theatres, concert halls, cinemas, and other venues should be maintained as long and as far as possible without having to compromise on infection protection.

For this purpose, the establishment professionally recognized and certified hygiene standards for cultural institutions makes sense. These should contain comprehensible and transparent criteria for infection protection that are relevant for the operator as well as for the staff and visitors. In addition, such a standard can serve as a basis for decision‐making for the federal states and their authorities responsible for infection protection.

The present overview study creates a basis for the intended hygiene standards and ensures that these correspond to the current state of science and research. In particular, it summarizes the current state of knowledge with regard to the technical requirements for drastically minimizing aerosol‐based infections at indoor cultural events and gives recommendations for various measures with a focus on ventilation, air distribution, air purification, and CO_2_ monitoring in cultural institutions with regard to the risk of infection by SARS‐CoV‐2. However, the influence of the vaccination status will not be considered. General information on the physical properties of bioaerosols, the risk of infection, and the use of face masks is briefly summarized, as this is necessary for understanding the available literature and the proposed measures.

As of March 2022, the recommendation for uniform hygiene and ventilation measures for cultural institutions under pandemic conditions and during normal operation, which was drawn up by an expert committee on the initiative of the German Federal Government, is already available [4], which can be accessed since August 10, 2022.

## METHODS

### Search strategy and data sources

The literature search followed the Preferred Reporting Items for Systematic Reviews and Meta‐Analyses (PRISMA) [5]. The search strategy was predefined by the demands of the German Federal Government. The aim was an overview study of the current state of knowledge in relation to the ventilation and logistical requirements for minimizing aerosol‐based infections at indoor cultural events. For this purpose, the relevant databases (SCOPUS, Web of Science [WoS], PubMed, and GoogleScholar [GS]) were selected and consulted. After the search terms had been chosen, article title, abstract, and keywords were queried in these databases. This was done independently by both authors. The respective results were then compared and merged.

### Inclusion and exclusion criteria

The abstract of all potentially interesting publications was screened. The main criteria for further consideration were dynamic studies on aerosols, including transmission and filtration, air and ventilation measurements in event rooms, as well as studies on people taking part in events. No studies were taken into account that deal with the economic situation of the cultural scene and venues or with the mental state of artists.

### Selection of studies

In the full‐text screening, both authors studied the publication content to refine the inclusion criteria. In particular, the literature cited in these publications was also considered. The decision to include or exclude a study was based on four criteria: (a) is the study relevant to answering the research question; (b) does the study provide reliable results; (c) was the study conducted under scientifically recognized conditions; and (d) can the results of the study be applied to the research question?

### Data and item extraction

The procedure for data extraction was defined in advance by the authors. The included items were as follows: information about the article (title, authors, year, DOI); information about the study (aim, focus, type, design, participants, and/or probands (if applicable), country); and outcome (main results, key conclusions, recommendations).

## RESULTS AND DISCUSSION

### General

**FIGURE 1:**
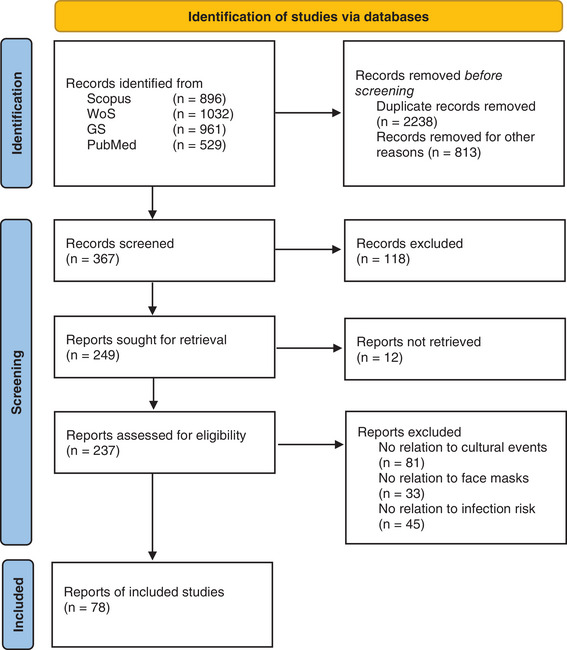
Preferred Reporting Items for Systematic Reviews and Meta‐Analyses (PRISMA) flow diagram according to Ref. [7] for a systematic review that included searches of different databases. GS, GoogleScholar; WoS, Web of Science

The keywords or keyword combinations “SARS‐CoV‐2,” “COVID‐19,” and “Indoor” resulted in around 3400 hits. With a refined keyword search (“cultural,” “event,” “concert,” “singing,” “choir,” “ventilation,” “face mask”), the number of potentially interesting publications was limited to 367, although only a small proportion directly related to the risk of infection at cultural events. As far as possible, the findings from other studies, for example, in school classrooms [6], were applied to the recommendations for this topic. The flowchart of the systematic literature selection according to PRISMA [7] is shown in Figure 1. Recommendations for the cultural sector were then derived on the basis of the available literature.

### Properties of bioaerosols

Today it is undisputed that the respiratory intake of bioaerosols that arise when breathing, coughing, speaking, and sneezing are one of the main transmission routes for SARS‐CoV‐2 viruses [8, 9]. From a physical point of view, an aerosol is a heterogeneous mixture of particles together with the gas or gas mixture surrounding them. In stable aerosols, the liquid or solid components are homogeneously distributed as floating particles. Everyone exhales liquid particles of different sizes. If a person is infected with a pathogen, these particles can also contain viruses or bacteria, which become airborne and are inhaled by other people.

Bioaerosols are accumulations of particles in the air, which contain fungi (spores, conidia, fragments of hyphae), bacteria, viruses, pollen, and their cell wall components and metabolic products (e.g., endotoxins and mycotoxins). Bioaerosols usually have aerodynamic diameters in the size range between 0.01 and 100 µm. SARS‐CoV‐2 is a membrane‐enveloped RNA virus with a size between 60 and 140 nm. In the air, however, it is usually surrounded by a watery envelope. Under laboratory conditions, viable viruses have been found to be detectable in airborne aerosols up to 3 h after release [10].

**FIGURE 2:**
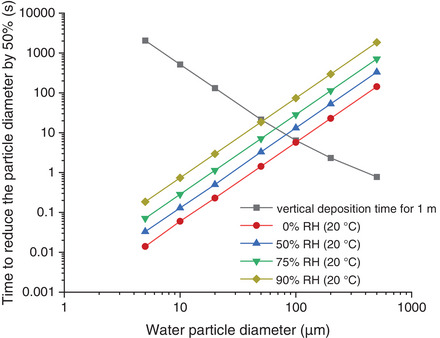
Time required for the diameter of pure water particles to decrease by 50% through evaporation at 20°C and various relative humidities. For comparison, the time required for a not shrinking particle to sink by 1 m in still air is also plotted (black curve).

Particles with a diameter of approximately 10 µm deposit within minutes and particles with a diameter of approximately 100 µm within seconds in still air. However, in real environments, the particles are also transported by air movement (advection and turbulent transport) and can therefore remain in the air for much longer. Aqueous particles, however, evaporate in the air depending on their size, temperature, and humidity. Figure 2 shows the time it takes for pure water particles of different sizes to halve their diameter through evaporation at 20°C and different relative humidities. For comparison, the time required for a not shrinking water particle to sink by 1 m in still air (black curve) is also plotted. The calculation was carried out according to common aerosol evaporation theory [11]. The diameter of a 100 µm particle is therefore halved by evaporation in dry air within 5 s and in humid air (90% relative humidity) in about 1 min. The evaporation time of a drop of water in still air is a function of the particle diameter, the particle density, the temperature of the particle and the ambient air, and the vapor pressure. This means that even large water particles do not necessarily fall to the ground but evaporate beforehand and remain in the air. At 20°C and 50% RH, the evaporation time of 90 µm particles is of the order of the falling time (see Figure 2). A very good overview of SARS‐CoV‐2 related aerosol physics can be found in the position paper of the Gesellschaft für Aerosolforschung (GAeF) [12].

### Properties of exhaled particles

The composition of the aerosol exhaled by humans depends on the respective activity. Speaking softly exhales fewer particles than speaking, singing, and shouting loudly. In addition, the ejection velocities are very different. The highest velocities are reached when coughing and sneezing. The number and size of particles released during human activities, such as breathing, speaking, coughing, and sneezing, have been well examined, and the results have been published in various studies.

In one study, it was found that particle emission during normal human speech is positively correlated with the loudness of vocalization, and emission rates of 1–50 particles per second were measured. Some individuals are “super‐emissive” and emit about an order of magnitude more particles than average [13].

The release of particles was measured during coughing and speaking using interferometric Mie imaging particle image velocimetry. The estimated total number of particles released ranged from 947 to 2085 per cough and 112 to 6720 for speaking. The geometric mean diameter of particles was 13.5 and 16.0 µm from coughing and speaking, respectively [14]. In another study, the size range from 45 to 150 µm was examined [15]. In vivo experiments showed that the number of exhaled particles varies over time and among subjects. Normal emitters have 14–71 bioaerosol particles/L in exhaled air, whereas superemitters release around 660–3230 bioaerosol particles/L [16].

It was reported that 50–1642 particles are released per cough reflex, while counting loudly led to significantly higher particle emissions [17]. The sizes of the particles were distributed between 1 and 1471 µm, with maxima at 1–3 and 26–55.6 µm [18]. The mass of particles when coughing was also determined [15]. Depending on their method, the authors found 22.9–85 mg. The particles were distributed over a range from 5 to 300 µm (maximums at about 35–150 µm). In another study [14], the particle size reached maximums at 6 (main peak) and 175 µm (secondary peak).

In sneezing fits, two patterns of size distributions were found as follows [19]: unimodal in 12 subjects and bimodal in 10 subjects. For the unimodal distribution, the maximum was 341.5–398.1 µm. The geometric mean was 360.1 µm, and the geometric standard deviation was 1.5 µm. For the bimodal distribution, the main peaks were at 72 and 386.2 µm with geometric standard deviations of 1.8 and 1.5 µm, respectively. Although only a very limited number of studies are available, the data show that speaking produces smaller particles than coughing and sneezing.

The following data were measured for the initial air velocity during coughing: 11.7 [14], 9.0 [20], and 15.3 m/s for men and 10.6 m/s for women [21]. The air velocity during speaking was much lower with 3.9 (men) [10], 4.07 (men), 2.31 m/s (women) [21], 1.08–1.56 m/s (men), and 1.53–1.64 m/s (women) [22]. The determined maximum airflow velocities were 4.5 m/s for sneezing, 1.4 m/s for nasal breathing, and 0.8 m/s for mouth breathing [23]. The sneezing velocity was lower than in other studies. This could be due to different techniques used in different studies and the limitation of the shadowgraph imaging technique, which requires a temperature difference between the exhaled puff and the ambient air. The air velocity decreases exponentially with distance from the mouth [24].

### Use of face masks

The use of personalized protective equipment (e.g., face masks) is a common measure worldwide against the transmission of viruses over the air. A distinction is made among three types: (a) particle‐filtering half masks (e.g., FFP2), (b) medical masks of type I or II (e.g., surgical masks), and (c) mouth and nose covers (e.g., fabric masks).

Several recent studies have measured the efficiency of face masks for trapping particles. When testing the effectiveness of common cloth masks in the laboratory, filtration efficiencies of 5%–80% for particles <300 nm and 5%–95% for particles >300 nm were found [25, 26]. However, these results are critically discussed, mainly due to the small pressure differences in this study compared to the pressure differences that occur under normal breathing conditions. This implies that the high filtration efficiency is mainly due to a very low air velocity of the particles [27, 28].

Other studies showed lower efficiencies for surgical and cloth face masks at a realistic pressure drop and air velocities [29, 30, 31, 32]. For example, a filtration efficiency of 5%–25% was found for common fabrics made of cotton, polyester, nylon, and silk [32]. In the same publication, filtration efficiencies of 6%–10% for polypropylene spunbond and 10%–20% for products paper based products are reported. Another study mentions a filtration efficiency of 17.4% for single layer cotton [29]. On the other hand, the N95/PN95 masks have shown significantly higher filtration efficiencies (>98%) [25].

Surgical face masks can also prevent virus transmission. Tests with a large number of individuals showed that the detection of influenza virus RNA in respiratory droplets and coronavirus RNA in aerosols is significantly reduced when wearing face masks [33]. In a model study, in which the filtration efficiency of a normal surgical mask for virus‐laden aerosols was assumed to be 50%, an infection probability of <1% could be achieved even in a confined space. Furthermore, it could be demonstarted that the wearing of surgical face masks during professional singing reduces the range of aerosol dispersion [34].

The filtration efficiency of different mask types was determined as a function of particle size by means of electrical mobility analysis [12], and it was concluded that FFP2 masks are most effective when used properly.

### Risk of infection and modeling tools

The risk of viral infection in an enclosed space depends on various factors. These include the concentration and infection rate of the virus, the distance between people, personal protective measures, and ventilation conditions. An effective process for reducing the concentration of particles in a room is dilution with clean and virus‐free air [35, 36]. Outdoors, dilution is constantly taking place through natural air movement. Indoors, dilution can be achieved through efficient ventilation. If possible, windows should be opened and air movement should be ensured as much as possible. The most effective way to do this is cross ventilation [37]. The ventilation time required depends on the size of the room, the number and size of the windows, and the temperature difference inside and outside. If necessary, the air exchange can be forced mechanically. Mobile air purifiers should only be used in exceptional cases.

The general risk of infection with SARS‐CoV‐2 viruses indoors has been addressed in various works. Different scenarios and activities (hospital, sports hall, public building, conference room, or auditorium) were compared [38], as was the risk of infection depending on the interpersonal distance, taking into account both the spatial and the temporal aspect [39]. In another work, 318 outbreaks were analyzed in terms of location and social contacts [40]. The risk of infection depending on the distance between people with and without a mask was also discussed [41]. It was concluded that if face masks are worn correctly, the distance between two people can be reduced to 0.5 m without increasing the risk of infection.

To calculate the risk of infection, many models require the so‐called quanta concentration in the room air [42, 43, 44]. A quanta describes the amount of viruses that, if inhaled by a person, will lead to an infection with a given probability. The assumption is usually made that the infected and infectious persons are in the room at the same time and that the quanta concentration in the ideally mixed room air corresponds to the equilibrium concentration for the entire period. The quanta concentration can be calculated from the quanta emission rate. In general, quanta values can only be roughly estimated.

The risk of infection in a well‐mixed room contaminated with virus‐carrying aerosols is often estimated using the Wells–Riley Equation (1). It is assumed that the risk of infection increases exponentially with the number of viruses inhaled. The Wells–Riley equation thus describes a classic one‐zone model without considering concentration gradients:

(1) $${\mathrm{AR}}_{\inf}=1-e^{-\frac{I\cdot q\cdot p\cdot \tau }{V_{R}}}$$

where AR_inf_ is the absolute risk of infection. This defines the probability that a person in the room will become infected under the given conditions. *I* is the number of infected people in the room, *q* is the quanta emission rate, *p* is the breathing volume flow of a person, *τ* is the time that an uninfected person stays in the virus‐polluted environment, and ${\dot{V}}_{R}$ is the supplied air volume flow into the room.

The Wells–Riley equation was modified several times in order to be able to estimate the risk of infection under different conditions and for different scenarios [45, 46, 47, 48, 49, 50, 51]. The Wells–Riley model has the disadvantage that rapid and ideal mixing is assumed. Therefore, the risk of infection is the same at any point in the room. The model was expanded for realistic room air flows, so that different infection risks can also be expected in different segments of the room [52]. However, this is associated with a corresponding amount of computational effort.

A complex spreadsheet model containing a number of modifiable environmental factors with relevant physiological parameters and environmental conditions was also developed [53]. This model takes into account the difference between everyday masks and FFP2 masks, different scenarios for air exchange, different virus mutations, so‐called superspreaders, and the change between quasi‐stationary and transient conditions. The properties of the aerosols and viruses can be modified in detail. A similar model was published on the website of the International Laboratory for Air Quality and Health (ILAQH) at Queensland University of Technology (QUT) in Brisbane, Australia. However, both models have the disadvantage, just like the Wells–Riley approach, that they assume ideal mixing of the air. As already mentioned several times, this is usually only the case with simple supply air/exhaust air systems in small rooms. A multi‐zone model for simulating the spread of infectious aerosols is available with the CONTAM software package [54].

A published guideline concept is based on models of airborne disease transmission [55], and the authors derive an upper limit for the cumulative exposure time. It is shown how this limit depends on the ventilation and air filtration rate, the dimensions of the room, the breathing rate, the respiratory activity and use of the face mask of its occupants, and the infectivity of the aerosols.

### Risk for infection at cultural events

**FIGURE 3:**
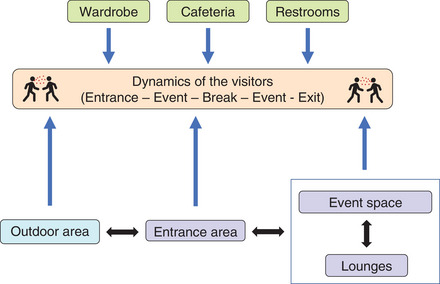
Dynamics of visitors during a cultural event

Only a few works have been published on this topic to date, which may be due to the fact that the possible scenarios are varied and of a complex nature. In addition, rooms in which cultural events take place can hardly be described with simple models. As a rule, complex approaches are necessary that take into account different concentrations in different areas (zones) of a building [54]. A typical dynamic scenario is shown in Figure 3.

The first phase of a cultural event is the arrival of the audience from admission to the start of the performance. If there are assigned seats, the audience can be taken directly there, but it is also usual to stay in an entrance area first. There is often catering or the possibility of handing in outer clothing. The second phase is the event itself. In the simplest case, it is a cuboid room with a stage at the end. All listeners are on one level, the artists sit, stand, or move in a slightly elevated position. Theaters usually have several levels (tiers) for the audience. The people are therefore at or above the level of the stage. Very large concert halls have grandstands, where the stage can also be in the middle. The third phase begins after the end of the performance and ends when all guests have left the venue. Here it is common for people to remain in the event hall or in the entrance area for a while. Phases 1 and 3 can be controlled by appropriate logistics. However, there are often breaks during which visitors may move around the event complex. The published studies are discussed later. Table 1 provides an overview.

**TABLE 1:** Overview of published studies relating to the risk of SARS‐CoV‐2 infection at cultural events

Event	Type of study	Ref.
Choir and other scenarios	Modeling study	[53]
Sociocultural events	Non‐randomized controlled study	[67]
Chorus rehearsal	Estimation of infectious quanta	[56]
Wind instruments	Risk of spreading infectious particles	[57]
Concert hall	Evaluation of the infection risk using mannequins	[58, 59]
Indoor sports and cultural events	simulation study to estimate the burden of disease under conditions of controlled epidemics	[60]
Mass gatherings	Effectiveness of public health measures	[68]
Mass sporting and cultural events	Use of interviews and questionnaires to describe the potential public health impact	[70]
Singing event	Evaluation of safer singing practices	[62]
Live concert	Randomized controlled trial of attendees	[63]
Indoor live concert	Screening of attendees for SARS‐CoV‐2 antigen before the event	[65, 66]
Indoor live events	Measurement of CO_2_ and viral RNA	[69]
Indoor live events	High resolution CO_2_ monitoring	[82]
Concert events	Assessment of SARS‐CoV‐2 transmission using contract‐tracing data	[61]
Carnival	Seroepidemiological observational study	[72]
Visitor's point of view	Online study with persons, who regularly attended events before the pandemic	[73]
Sport events	Assessment of the suitability and feasibility of a hygiene concept	[71]

The infection risk for four different indoor situations (office, classroom, choir, and reception) and different scenarios (active and passive ventilation, type of masks, and filters) was modeled [53]. It was assumed on the one hand that the exhaled number of viruses corresponds to the usual magnitudes (standard event) and on the other hand that the virus output is increased (superspreading event). In summary, choral singing caused the highest risks.

The spread of SARS‐CoV‐2 viruses at a church event was examined [56]. A total of 61 people met to sing in a choir, 53 of whom were infected. Precautions were taken during the rehearsal, including the use of hand sanitizer, no hugs, and no handshakes. All 120 chairs were arranged by three people who arrived early, and members sat in their usual chairs. Lateral spacing between chair centers (and hence nose‐to‐mouth spacing) was ∼0.75 m, whereas front spacing between rows was ∼1.4 m.

A total of 14 wind instruments were analyzed in a typical environment for classical musicians [57]. This was done qualitatively by making air currents visible while playing and then quantitatively by measuring the air speed at three distances (1, 1.5, and 2 m) from the respective instrument. The measurements took place on musicians of the Bamberg Symphony Orchestra in their concert hall. The results show that while playing, no airflow was measurable beyond a distance of 1.5 m for any wind instrument, for brass instruments from the bell or for woodwind from the mouthpiece, vents or bell, regardless of volume, pitch or what was played. The air speed when playing corresponded to the value of 1 m/s that is usual in hall‐like rooms. In the case of woodwind instruments, alto flute, and piccolo, clear air movements could be observed near the mouthpiece.

The spread of aerosols in the audience area of several concert halls was experimentally investigated in order to assess their airway and thus the risk of spreading an infection with SARS‐CoV‐2 [58]. For this purpose, a dummy was used that emits simulated human breath with aerosols (mean diameter 0.3 µm) and CO_2_ with a horizontal exhalation speed of 2.4 m/s, measured 10 cm in front of the mouth. Aerosol and CO_2_ concentration profiles were determined using sensors placed around the dummy. No significant accumulation of aerosols and CO_2_ was detected at adjacent seats if displacement ventilation was provided under each seat, which enabled a local fresh air vertical flow of at least 0.05 m/s, if the air exchange rate was higher than 3 h^−1^ and the dummy wearing a surgical face mask. These experiments are described in more detail in another publication by the same authors [59]. The different results depending on wearing or not wearing a face mask are also demonstrated here. Wearing a mask significantly reduces the risk of infection for people sitting in front of an emitter.

The risk of particle and aerosol transmission of SARS‐CoV‐2 during a mass event was investigated under three different hygiene practices. The data were used in a simulation study to estimate the resulting disease burden under controlled epidemic conditions [60]. The results show that the average number of measured direct contacts per visitor was nine people and that this can be significantly reduced through appropriate hygiene practices. A comparison of two ventilation variants with different air exchange rates and different air flows showed that the system with the worst performance caused a tenfold increase in the number of people exposed to infectious aerosols. The overall burden of infection from indoor mass accumulations therefore depends significantly on the quality of the ventilation system and hygiene practices. Assuming an effective ventilation system, the authors believe that indoor mass gatherings with proper hygiene practices have very little, if any, impact on the spread of the epidemic.

SARS‐CoV‐2 infection chains were identified at several small concert events in the Osaka area, Japan [61]. The infected persons were identified by evaluating contact tracing data. It also became clear that the spread of the virus from one event to another was facilitated by infected people attending multiple events.

The risk of infection with SARS‐CoV‐2 at singing events was discussed [62]. The authors conclude that the risk can certainly be reduced by taking suitable measures, but that risks cannot be completely eliminated. Every community of singers and artists must do everything possible to mitigate the risk as much as possible and then decide whether this risk reduction of SARS‐CoV‐2 transmission is sufficient to resume the singing activity under consideration of all the discussed factors. From today's perspective, the statement sounds trivial but leads to the conclusion that there can be no generally applicable recommendation. The measures must always be adapted to the respective situation.

SARS‐CoV‐2 infection rates among those attending a major live concert on May 29, 2021 in Paris were compared to those who were not present at that concert [63]. The effectiveness of mask wearing was also rated. The results did not show a significantly increased risk of SARS‐CoV‐2 transmission among participants compared to nonparticipants. In this context, it was commented that in May 2021, most SARS‐CoV‐2 infections in France were caused by the alpha variant [64]. With the significantly more contagious delta variant (note: the delta variant refers to the time of writing the article), the transfer of the results from Paris [63] to other conditions is not necessarily given.

Antigen testing was used to study transmission rates during an indoor live music concert with 5000 people in Barcelona [65, 66]. Singing and dancing were allowed, and no minimum distance was required. Six people who attended, none of whom were vaccinated, were diagnosed with COVID‐19 within 2 weeks after the concert.

Another study showed that attendance to events that involved social interaction with a certified digital pass was not associated with an increased rate of SARS‐CoV‐2 infection compared to a control group [67]. Logistical problems were infrequent and easy to solve, the participants’ overall opinion on the accreditation process was satisfactory.

The risk of infection at mass events was also dealt with. A review considering 11 studies found that implementing measures can reduce the risk of SARS‐CoV‐2 transmission [68]. However, it is unlikely that this risk can be completely eliminated. All studies followed a multipronged, multi‐measure approach. This seemed more effective than relying on a single measure. The number and intensity of measures implemented varied by study, with many being resource intensive. There is currently limited evidence for the effectiveness of measures to prevent SARS‐CoV‐2 transmission at mass gatherings. With such events resuming, continued known mitigation measures are required to limit the risk of transmission, as well as ongoing research and monitoring to assess the potential impact of these events on the wider population and healthcare system.

An extensive study of cultural events was conducted in England [69]. SARS‐CoV‐2 RNA was detected on a small number of surfaces at very low copy numbers, which are unlikely to pose an infection risk. The respective ventilation conditions in the halls were assessed based on the CO_2_ concentration, taking into account the ventilation strategies and occupancy levels. Although ventilation conditions were generally good, some seating areas were exposed to poor mixing of air. Overall, the authors conclude that most theaters pose a low risk of long‐range infection with SARS‐CoV‐2.

Contact tracing data were collected through telephone interviews and online questionnaires to investigate the different numbers of infections at sporting events with comparable numbers of visitors [70]. These were attributed to socioeconomic factors, different safety awareness among the visitors, and their different dynamics of movement.

The suitability and feasibility of a hygiene concept was assessed during a series of indoor sporting events [71]. The increasing spectator numbers consolidated and optimized hygiene concept‐related processes and demonstrated a high level of spectator compliance with the hygiene measures.

During carnival in a small German town, a SARS‐CoV‐2 super‐spreading event was reported [72]. Due to quickly imposed lockdown measures and the resulting relatively closed community, this town has been considered a good model for studying infection dynamics. A 7‐day observational study was conducted to collect information and biological samples from a randomized, household‐based study population.

Another important point concerns the question of how visitors prepare for participation in an event, what preventive measures they take and what expectations they have about the organizer. This aspect was examined as part of an online study [73]. The use of disinfectants was mentioned as the most important element of containment, followed by transparent information on the hygiene strategy, reduced crowds, optimized ventilation, body temperature measurement at the entrance, negative SARS‐CoV‐2 test, and filling out a health questionnaire. Forgoing breaks and meals were seen as less important.

Analyses of the situation in a poorly ventilated courtroom [74] showed parallels to a cultural event. The hearing took place on a kind of stage, with different people interacting with each other verbally. If necessary, the seats for spectators could be separated from this stage. It was found that in such situations and without further protective measures, the risk of infection is particularly high.

### Criteria for ventilation conditions

**FIGURE 4:**
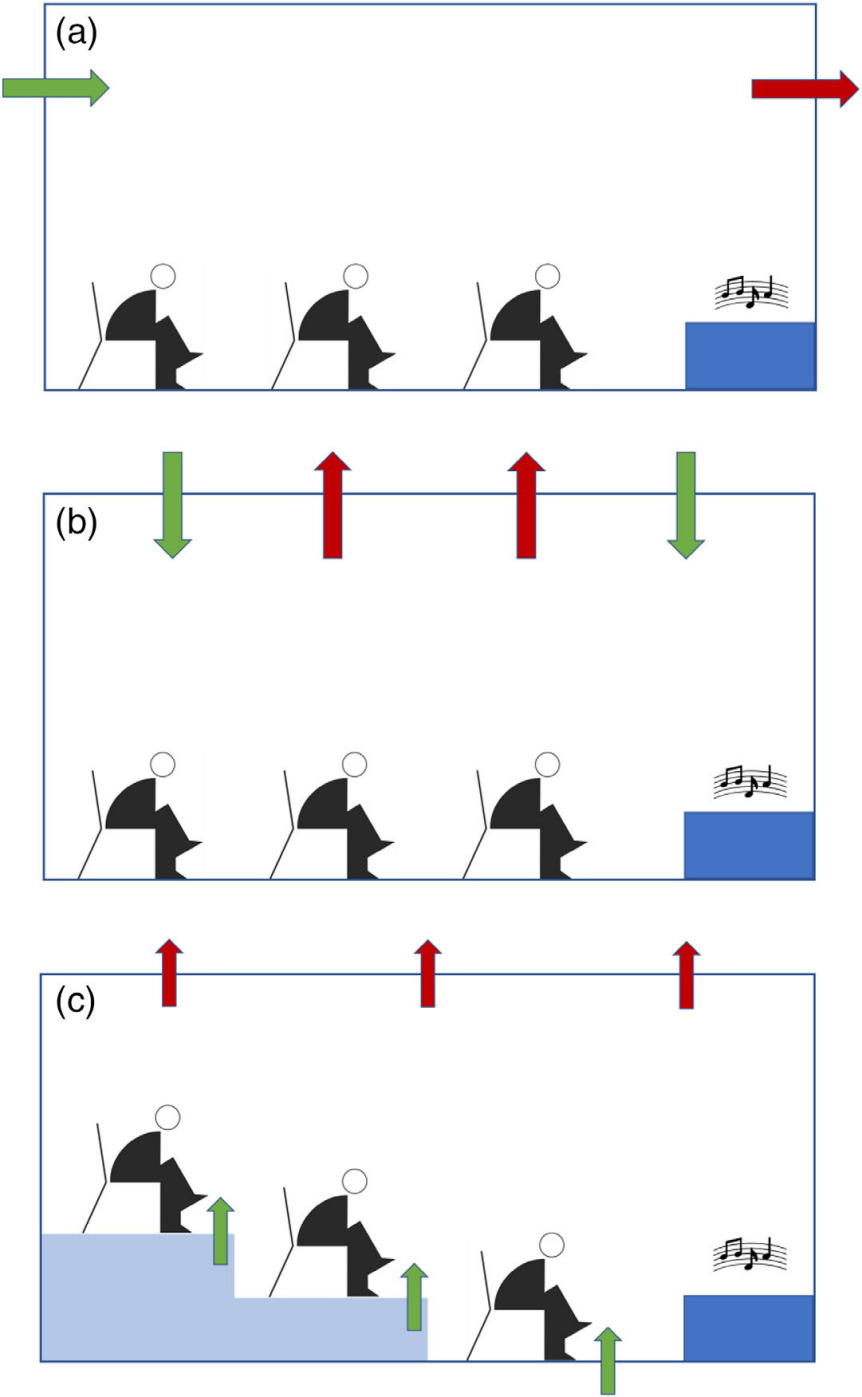
Different types of air supply and air distribution during a cultural event. (a) Simple ventilation by fans; (b) ventilation system with inlet and outlet in the ceiling area; (c) individual ventilation directly at the seat and outlet in the ceiling area. The green and red arrows indicate air inlet and outlet, respectively.

Efficient air supply and distribution through mechanical ventilation units in a building is challenging and many technical solutions exist [75, 76]. However, the big problem with cultural and sporting events is the complexity of the halls and arenas. Only in a few cases will simple air supply, as shown in Figure 4a, be sufficient. It must then be assumed that the air is completely mixed so that, as in school classrooms, the risk of infection can be assessed according to the Wells–Riley Equation (1).

Larger halls often have multiple air inlets and outlets at the ceiling as shown in Figure 4b. Individual air supply makes sense for complex seating arrangements with several levels (see Figure 4c). However, this is only possible with full seating.

**FIGURE 5:**
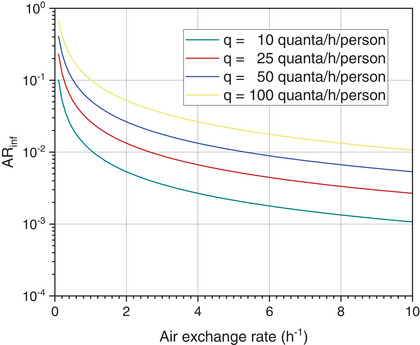
Absolute risk of infection AR_inf_ according to the Wells–Riley equation in dependence of the air exchange rate. The calculation was carried out with Equation (1) for different quanta, see text for parameters and colors of the curves.

The example shown in Figure 5 is certainly at the limit of what is possible with the Wells–Riley equation. A room volume of *V* = 1260 m^3^ (18 m × 14 m × 5 m) was selected with the presence of *N* = 96 attendees and one infected person (*I* = 1) in the room. A respiratory volume flow of *p* = 0.54 m^3^/h (calculated for an adult person with very little exertion [77]) and a residence time of *τ* = 2.5 h were assumed per person. The air exchange (AER) was between 0.1 and 10 h^−1^, and the viral (infectious) release rate was *q* = 10 (green), 25 (red), 50 (blue), and 100 (yellow) quanta/h per person:

(2) $$\mathrm{AER}=\frac{1000\cdot N\cdot {\dot{Q}}_{{\mathrm{CO}}_{2}}}{(C_{{\mathrm{CO}}_{2}}\left(\infty \right)-C_{{\mathrm{CO}}_{2}\left(\mathrm{ambient}\right)})\cdot V}$$

It is further assumed that each person exhales $\dot{Q}$ = 20 L of CO_2_ per hour and that the background concentration of CO_2_ (ambient) is 400 ppm. According to Equation (2), the air exchange required for a CO_2_ steady‐state concentration of <2000 ppm is 1 h^−1^. In order to permanently fall below a CO_2_ concentration of 1000 ppm, as recommended, for example, at schools [78] and rooms with many people inside [79], an air change of 2.6 h^−1^ would be necessary. Assuming that the distance regulation is observed (each person has 1.5 m × 1.5 m space), the theoretical absolute risk of infection AR_inf_ with an air change of 2.6 h^−1^ and the highest q is still 0.04. In practice, these values are likely to be far too optimistic. Carbon dioxide has a higher molecular weight and a higher density than nitrogen and oxygen and unless the air is well mixed it will tend to accumulate near the ground. This requires very good mixing conditions, which cannot be achieved with natural ventilation, and at the same time, an increased air exchange to prevent contaminated air from being distributed in the hall.

Regardless of pandemics, it is generally important to ensure good ventilation conditions in rooms. This applies to cultural events as well as to school classrooms and means of transport. Corresponding measurements require high temporal resolution and automation, which is possible with real‐time monitoring devices [78, 80]. This usually excludes the direct detection of viruses, so other parameters must be used. With regard to hygienic aspects, the carbon dioxide concentration in the room air serves as an indicator for air deterioration caused by human activity. The concept of the German Committee on Indoor Air Guide Values (AIR—formerly Ad hoc AG) [79] considers CO_2_ concentrations of less than 1000 ppm (0.1% by volume) to be hygienically harmless and CO_2_ concentrations greater than 2000 ppm to be hygienically unacceptable.

In rooms with high occupancy, the carbon dioxide concentration can serve as an indication of good or bad ventilation. This means that information can be provided quickly and easily as to whether and when ventilation is necessary. However, this does not mean that a CO_2_ concentration of less than 1000 ppm generally protects against infection with SARS‐CoV‐2. Conversely, CO_2_ concentrations significantly or permanently higher than 1000 ppm indicate inadequate ventilation management with a potentially increased risk of infection. Inexpensive and reliable sensors are available for continuous carbon dioxide measurement. Their suitability could be demonstrated, for example, by measurements in local public transport [80, 81]. Moreover, high‐resolution measurements (determination of CO_2_ in intervals of about 1 min) enable the situation at live events to be monitored and are thus useful for a quick assessment of the distribution and effectiveness of the ventilation [82]. In a Spanish publication [81], the criterion is 800 ppm carbon dioxide (which is applied e.g., in European standards for the regulation of indoor ventilation systems in nonresidential buildings) instead of 1000 ppm. Other authors [83] also assess the air quality at singing events on the basis of 800 ppm CO_2_. A four‐stage CO_2_ concept between <950 (high IAQ) and >1750 ppm (low IAQ) is proposed in an English publication [83]. Overall, it can be concluded that controlling the supply and exhaust air based on CO_2_ measurements is an effective preventive measure against airborne infections at cultural events [84].

The determination of the particle concentration, especially PM_10_ and PM_2.5_, is also an important aspect for evaluating the indoor air quality [85, 86]. However, PM is not very suitable for checking ventilation requirements, as the dynamics of particles are already high due to the movement of people. Some of the particles are introduced via the outside air, some via clothing. Another parameter that can be measured easily and quickly is the sum of volatile organic compounds as the so‐called TVOC_PID_ value [87]. TVOC stands for “Total Volatile Organic Compounds” [88] and PID for the measurement method using photoionization. However, TVOC_PID_ is not directly linked to the population density and the breathing rate of people, so that this parameter should at best be used in addition to the CO_2_ concentration.

### International recommendations and guidelines for cultural events

In June 2021, the European Commission published EU guidelines for the safe resumption of activities in the cultural and creative sectors [3]. Testing events organized in the EU have shown that few COVID‐19 cases have been linked to transmission at or around cultural events. Particular requirements for participation and monitoring of data in the follow‐up of these trials were essential for a safe organization. The Commission has developed population‐related, individual‐related, and event‐related indicators and recommends that the member states take these into account when opening up cultural activities.

The initiative of the German Federal Government has already been reported in an earlier chapter [4].

The Austrian guidelines of the Medical University of Vienna are intended to define medical framework conditions for the specific practice of cultural institutions or medical measures that are aimed at ensuring that visitors to a cultural institution are not exposed to a higher risk than with other contact with people in public space [89]. The focus here is on the complex of questions of the game operation with audience/visitors. Questions about presenting artists (actors, musicians/orchestras) as well as technical and administrative staff are addressed but not elaborated on.

In France, the Haut Conseil de la Santé Publique has published various texts on how to deal with the SARS‐CoV‐2 virus at cultural events [90].

In the United Kingdom, the Events Research Programme (ERP) aims to examine the risk of transmission of COVID‐19 from attendance at events and explore ways to enable people to attend a range of events safely [91].

In March 2022, the Australia Council of Arts published the “Audience Outlook Monitor” with recommendations on how to behave when attending live events [92].

In the United States, the Centers for Disease Control and Prevention (CDC) issues guidelines for small and large gatherings and events, which are updated regularly [93].

## CONCLUSION AND RECOMMENDATIONS

It was shown that cultural events take place under very different conditions. It is therefore practically impossible to predict the exact risk of infection for a specific situation or to derive general rules that go beyond the known measures of vaccination, testing, masks, and distancing. In addition, there are a number of important factors that cannot be controlled or regulated. In European countries, the vaccination rate varies greatly from region to region, and counterfeit vaccination and test certificates must also be expected. Furthermore, the emotions of the viewers must be taken into account. It is certainly difficult not to move or not to sing along in an appealing concert. For very small events with approximately 50 participants and seated people, the criteria for school classrooms can be applied if necessary. Such simple scenarios can be estimated using the Wells–Riley equation or similar models. Adequate mixing of the air can already be achieved here with simple ventilation systems.

Very large halls with up to 20,000 seats for visitors are just as difficult to model as architecturally complex medium‐sized concert halls. However, the event itself is only one aspect. Equally important are questions of guiding people when entering and leaving and the connection to other premises (VIP lounges, cafeteria, etc.). Whatever measures are taken by the organizers, successful implementation always requires a certain understanding and awareness of the situation. All of these points make it difficult to collect meaningful data at a cultural event, with uncertainties increasing as the number of participants increases. In addition, the infectious persons are not necessarily recorded representatively in test series on site. It is less likely that people who are generally critical of vaccinations and tests will take part in a study on site.

In general, it is not possible to base a risk analysis on just one single parameter. For example, the distance control or staggered seating arrangements will be of little use if the ventilation in the hall is insufficient. Table 2 gives a selection of the main influencing variables. The most important point is definitely air exchange. Sufficient uncontaminated fresh air must be supplied to the room, which is appropriate for the room size and the number of people in the room. The fresh air must be effectively distributed in the room. This requires effective concepts for both the introduction and the air flow, which are adapted to the individual room or hall.

**TABLE 2:** Parameters that should be considered when holding a cultural event under pandemic conditions

Factor/measure	Implementation/performance
Room type	Floor space and room height Spatial geometry of the room Number of levels Arrangement of the levels (if several exist) VIP lounges available? Position of stage (if any)
Dynamics before, during, and after the event	Distance rules at the entrance and exit Duration of the event Breaks Catering
Type of performance	Reading, lecture, movie Play (language only) Operetta, opera Concert (only instrumental or with vocals)
Position of the audience	Standing Sitting (side by side, staggered, others)
Reactions of the audience	Sitting or standing still Moving or dancing Singing along
Vaccination status and behavior of the audience	Vaccinated (1×, 2×, 3×, and so on) Negative test Masks (FFP2, other medical masks) Use of disinfectants
Room ventilation	Natural ventilation (opening windows and doors) Fans (without heat exchanger) Ventilation system (number and position of air inlets and outlets) Air volume flow and proportion of recirculated air Personalized supply air Connection of the ventilation system to other rooms, e.g., B. VIP lounges
Air cleaning measures	None Integrated into the ventilation system Mobile air purifiers Spraying of active substances

Simple systems, such as an air inlet and an air outlet, will only make sense in relatively small classroom‐sized rooms. More complex systems are required for events with many participants. In large arenas, it makes sense to individually adjust the air supply to the room or room use (event hall, lounges, cafeteria, entrance area, etc.). In addition, measuring the carbon dioxide concentration at several points in the room is a very helpful parameter for assessing the need for ventilation and airflow. It has already been mentioned that there is hardly any experience and data on the risk of infection with SARS‐CoV‐2 viruses at cultural events, which makes it even more difficult to develop the necessary measures.

The use of mobile air cleaners and similar disinfecting measures at cultural events will only be possible and meaningful in exceptional cases. Uhde et al. [94] provided an overview on this topic with reference to school classrooms. It is important that a mobile air purifier cannot replace the necessary fresh air supply.

Finally, it must be clearly pointed out once again that any recommended action for hygiene and ventilation measures in cultural institutions under pandemic conditions may reduce the risk of infection but cannot completely rule it out. The measures planned by the organizers can only relate to possible exposure to infectious aerosols via the airway. It has to be assumed that the visitors protect themselves individually and also keep the recommended or specified distances. The latter point in particular is likely to be essential, as analyzes of passenger flows in local and long‐distance public transport have shown.

## AUTHOR CONTRIBUTIONS

*Conceptualization; funding acquisition; investigation; methodology; project administration; validation; visualization; writing‐original draft*: Tunga Salthammer. *Conceptualization; methodology; validation; writing—review; and editing*: Heinz‐Jörn Moriske. Both authors approved the final draft.

## CONFLICTS OF INTEREST

The authors declare that they have no conflicts of interest.

## Data Availability

Data sharing is not applicable to this article as no new data were created or analyzed in this study.
